# Compartmentalization of GABA Synthesis by GAD67 Differs between Pancreatic Beta Cells and Neurons

**DOI:** 10.1371/journal.pone.0117130

**Published:** 2015-02-03

**Authors:** Jamil Kanaani, Chiara Cianciaruso, Edward A. Phelps, Miriella Pasquier, Estelle Brioudes, Nils Billestrup, Steinunn Baekkeskov

**Affiliations:** 1 Departments of Medicine and Microbiology/Immunology, Diabetes Center, University of California San Francisco, San Francisco, California, United States of America; 2 Institute of Bioengineering, School of Life Sciences, École Polytechnique Fédérale de Lausanne, Lausanne, Switzerland; 3 Cell Isolation and Transplantation Center, Department of Surgery, Geneva University Hospitals and University of Geneva, Geneva, Switzerland; 4 Institute of Biomedical Sciences, University of Copenhagen, Copenhagen, Denmark; University of Lille Nord de France, FRANCE

## Abstract

The inhibitory neurotransmitter GABA is synthesized by the enzyme glutamic acid decarboxylase (GAD) in neurons and in pancreatic β-cells in islets of Langerhans where it functions as a paracrine and autocrine signaling molecule regulating the function of islet endocrine cells. The localization of the two non-allelic isoforms GAD65 and GAD67 to vesicular membranes is important for rapid delivery and accumulation of GABA for regulated secretion. While the membrane anchoring and trafficking of GAD65 are mediated by intrinsic hydrophobic modifications, GAD67 remains hydrophilic, and yet is targeted to vesicular membrane pathways and synaptic clusters in neurons by both a GAD65-dependent and a distinct GAD65-independent mechanism. Herein we have investigated the membrane association and targeting of GAD67 and GAD65 in monolayer cultures of primary rat, human, and mouse islets and in insulinoma cells. GAD65 is primarily detected in Golgi membranes and in peripheral vesicles distinct from insulin vesicles in β-cells. In the absence of GAD65, GAD67 is in contrast primarily cytosolic in β-cells; its co-expression with GAD65 is necessary for targeting to Golgi membranes and vesicular compartments. Thus, the GAD65-independent mechanism for targeting of GAD67 to synaptic vesicles in neurons is not functional in islet β-cells. Therefore, only GAD65:GAD65 homodimers and GAD67:GAD65 heterodimers, but not the GAD67:GAD67 homodimer gain access to vesicular compartments in β-cells to facilitate rapid accumulation of newly synthesized GABA for regulated secretion and fine tuning of GABA-signaling in islets of Langerhans.

## Introduction

In mammals, two highly homologous non-allelic isoforms of the enzyme glutamate decarboxylase (GAD), GAD65 and GAD67, synthesize the major inhibitory neurotransmitter GABA from glutamate [1, 2], thereby controlling its cellular levels. GAD and GABA are expressed in GABA-ergic neurons in the central nervous system [1] and in insulin-producing β-cells in the islets of Langerhans [3] where secreted GABA functions as a growth factor for cells and as a paracrine [4, 5] and autocrine signaling molecule [5, 6] involved in regulation of hormone secretion [7]. GAD67 binds the coenzyme pyridoxal 5-phosphate (PLP) tightly and is constitutively active, producing ~90% of basal GABA levels in the brain. In contrast, GAD65 oscillates between an active PLP-bound holoenzyme and an inactive apoenzyme [8, 9]. The evidence from GAD65 and GAD67 knockout mice is consistent with a mechanism whereby GAD67 provides most of the basal levels of GABA for inhibitory neurotransmission, whereas transiently activated GAD65 synthesizes GABA in response to sudden increases in GABA demand, so as to fine-tune GABA-ergic synaptic function [10, 11]. GAD65’s unique membrane anchoring properties and ability to rapidly cycle between synaptic vesicle membranes in axon termini and a depot in Golgi membranes via a palmitoylation/depalmitoylation mechanism [12, 13] is likely an integral part of the rapid GABA response mediated by GAD65 synthesis [14, 15].

GAD65 and GAD67 are highly homologous proteins, except in their N-terminal regions that mediate distinct membrane anchoring properties of the two isoforms [2]. Both GAD65 and GAD67 are synthesized as soluble hydrophilic molecules. GAD65 undergoes a set of intrinsic post-translational hydrophobic modifications in the N-terminal domain [16, 17]. The first set of hydrophobic modifications is reversible and results in a peripheral association with the cytosolic face of ER and Golgi membranes [16–18]. This form can cycle between the cytosol and ER/*cis*-Golgi membranes until a double palmitoylation of cysteines 30 and 45 [19] by the palmitoyl transferase HIP14 [20], results in anterograde trafficking through the *trans*-Golgi network to vesicular compartments and a final destination in synaptic vesicles in axon termini [12, 18, 24]. GAD67, in contrast, remains hydrophilic [22], and instead acquires–by two different mechanisms–the capability for membrane anchoring and trafficking to Golgi membranes, cytosolic vesicles, and presynaptic nerve terminals in neurons. The first mechanism involves heterodimerisation of GAD67 and GAD65 through their middle domains, and consequent piggy-backing of GAD67 onto GAD65’s membrane trafficking pathway [22, 23]. The second mechanism is independent of GAD65, involving the N-terminal domain of GAD67, which mediates piggy-backing of GAD67 onto another membrane-binding moiety [23]. The importance of the GAD65 independent mechanism was shown by analyses of neurons of GAD65-/- mice, which revealed that mouse GAD67 is firmly membrane anchored and efficiently transits to presynaptic clusters in the absence of GAD65, despite lacking intrinsic hydrophobicity [22, 24]. The GAD65-independent membrane anchoring mechanism of GAD67 may explain how GABA synthesized by GAD67 suffices for normal brain development and satisfies most requirements for GABA-ergic neurotransmission in adult GAD65-/- mice.

While neurons in different parts of the brain typically express both isoforms of GAD, human β-cells only express GAD65, mouse β-cells express GAD67, and lack detectable expression of GAD65, whereas rat β-cells express both isoforms of the enzyme [25–27]. The functional consequence of this inter-species variation in β-cell GAD isoform-expression is unknown.

In addition to serving as an intracellular source of fuel in β-cells [28], GABA is released from rat β-cells by regulated Ca^+2^-dependent exocytosis during membrane depolarization in response to physiological voltages [4]. Consistent with this finding, the islets of Langerhans express components required for GABA signaling. Thus, GABA_A_ receptors are detected in α, β, and δ-cells of human islets [29] and in α- [30] and β-cells in murine islets [31, 32]. GABA_B_ receptors have been detected in human [29] and mouse β-cells [33] and in rat α- and β-cells [4]. Furthermore, the plasma membrane GABA transporter GAT3 is expressed in α- and β-cells [34]. Finally, the finding that the vesicular GABA/glycine transporter VGAT is expressed at high levels in α-cells and either low or non-detectable levels in β-cells [34–36] suggests that a distinct β-cell vesicular transporter is yet to be identified. The detailed mechanism of GABA signaling through GABA_A_ and GABA_B_ receptors in islets and the effects on hormone secretion is not fully clarified, and the results of independent studies are often conflicting. For instance, while there is a general consensus that GABA secreted by β-cells inhibits glucagon secretion by α-cells via GABA_A_ receptors [37–39], different studies of the effect of GABA receptor signaling on somatostatin secretion, suggest inhibition, stimulation, or no effect [29, 37, 40, 41]. GABA’s effect on insulin secretion varies with the glucose concentration and species, and depends on whether signaling is through GABA_A_ or GABA_B_ receptors. While signaling via GABA_A_ receptors is stimulatory for insulin secretion [29, 31], signaling via GABA_B_ receptors appears to be inhibitory [4, 5, 42].

GAD65 has distinguishing features from GAD67 that make it differentially susceptible to becoming a target of autoimmunity in the two cell types that express it, neurons and islet β-cells. Autoimmunity to GAD65 in β-cells is associated with β-cell destruction and development of Type 1 diabetes [43] while autoimmunity to GAD65 in GABA-ergic neurons is associated with development of a rare neurological disorder, Stiff Person Syndrome [44].

Although the initial studies of the hydrophobic forms and membrane anchoring of endogenous GAD65 were carried out in islets of Langerhans [16, 17], the studies of the role of palmitoylation and subcellular trafficking, which required high resolution confocal microscopy, were performed in neurons, for which suitable techniques were available [12, 18, 22, 23]. In order to similarly elucidate the membrane trafficking and fate of GAD65 and GAD67 in β-cells, we have now developed a method to grow and transfect primary cultures of islet cells in order to study the GAD isoforms in β-cells by high-resolution confocal microscopy. This approach has revealed that GAD65 membrane trafficking is similar to neurons, but that β-cells lack the neuronal mechanism for GAD65-independent mechanism of GAD67 membrane anchoring [23]. Therefore, in β-cells, GAD67 can only be anchored to membranes by dimerization with GAD65 and the β-cell is distinct from neurons in only expressing soluble GAD67 in the absence of GAD65.

## Materials and Methods

### Antibodies

The following primary antibodies were used for immunostaining of Western blots: mouse monoclonal antibody to GFP (Covance, Richmond, CA); the 1701 rabbit polyclonal antibody, which recognizes GAD65 and GAD67 equally well on Western blots [25]. The following primary antibodies were used in immunofluorescence experiments: chicken anti-GFP polyclonal antibody (Millipore); mouse monoclonal antibody against the Golgi matrix protein of 130 kDa (GM130; BD Biosciences, Palo Alto, CA); guinea pig anti-insulin polyclonal antibody (Millipore); mouse monoclonal (ascites) antibody against GAD65 isoform (GAD6, a gift from Dr. D. Gottlieb, Washington University, Saint Louis, MO, [45]; rabbit polyclonal antibody against GAD67 isoform (K2, Millipore); a mixture of GAD65-specific human monoclonal antibodies MICA 2, 3, and 6, derived from a newly diagnosed type 1 diabetic patient (a gift from Dr. W. Richter, University of Heidelberg, Heidelberg, Germany) [46]; rabbit anti-synaptophysin polyclonal antibody (Life Technologies, Carlsbad, CA); rabbit polyclonal antibody against the Golgi marker protein giantin (Abcam, Cambridge, MA); Living Colors DsRed rabbit polyclonal antibody raised against DsRed-Express that recognizes mCherry (Clontech, Palo Alto, CA); and chicken anti-calreticulin (ER lumen marker) polyclonal antibody (Abcam). A rabbit anti-NAP22 polyclonal antibody (Millipore) was used for immunostaining of Western blots and immunofluorescence of fixed cells. The following secondary antibodies were used in immunofluorescence experiments: Cy3-conjugated donkey anti-mouse, anti-rabbit, or anti-human IgG; Cy3-conjugated goat anti-guinea pig IgG (all from Jackson ImmunoResearch Laboratories, West Grove, PA); Cy3-conjugated goat polyclonal antibody to chicken IgY (Abcam); Alexa Fluor 405 anti-mouse IgG; Alexa Fluor 488 goat anti-mouse, anti-rabbit, or anti-chicken IgG; Alexa Fluor 568 goat anti-rabbit; Alexa Fluor 633 goat anti-chicken IgG (all from Life Technologies).

### Islet and neuron isolation, cell culture, and transfection

Neonatal rat islets were isolated from 4–5-days-old Sprague Dawley rat pups (Charles River, Wilmington, MA) as previously described [47] except using collagenase P (Roche Diagnostics, Indianapolis, IN) digestion and Histopaque 1119 (Sigma Aldrich, St. Louis, MO) density gradient purification. Mouse islets were isolated from 3–5-weeks-old C57BL/6 mice (Charles River), as previously described [48]. Human islets were obtained from the European Center for Islet Transplantation (ECIT) Islets for Basic Research Program. Isolated islets were pre-cultured at 37°C in 5% CO_2_ for 1–2 days in RPMI 1640 medium supplemented with glutamax, 100 U/ml penicillin, 100 μg/ml streptomycin, and 10 mM HEPES (all from Life Technologies). Islets were hand-picked, washed once with Ca^2+^/Mg^2+^-free Hank’s BSS, and dispersed into single cells by a brief incubation with 0.05% trypsin-EDTA in a Ca^2+^/Mg^2+^-free medium using a mild mechanical disruption. The dispersed islet cells were seeded (25,000–30,000 cells/well) on coverslips coated with extracellular matrix (ECM) in four-well culture plates (Biological Industries, Kibbutz Beit Haemek, Israel) in RPMI-1640 medium supplemented with glutamax, 2% human serum (Lonza, Walkersville, MD), 100 U/ml penicillin, 100 μg/ml streptomycin, and 10 mM HEPES. The single cells were allowed to adhere for 3–5 days before experiments. Single islet cells were transfected for 5 h with either Lipofectamine 2000 or Lipofectamine Plus reagents according to the manufacturer’s instructions (Life Technologies) with similar transfection efficiency of ~0.1% (~25 transfected cells/25,000 cells). For indirect immunofluorescence analysis, cells were fixed 24–48 h after transfection with 4% paraformaldehyde (EM grade; Electron Microscopy Sciences, Hatfield, PA). Rat insulinoma INS-1 cells [49] and mouse insulinoma MIN6 cells [50] were obtained from Dr. John Hutton (Barbara Davis Diabetes Center, Denver, Colorado) and cultured in RPMI 1640 medium and DMEM medium respectively with high glucose (25 mM glucose). For indirect immunofluorescence analysis, cells were cultured on glass coverslips coated with poly-D-lysine and fibronectin in 24-well plates. INS-1 and MIN6 cells were transfected for 5 h with Lipofectamine Plus reagent according to the manufacturer’s instructions (Life Technologies) with a transfection efficiency of ~40%.

Primary hippocampal neuronal cultures were prepared from either embryonic day 18/19 rats [51] or postnatal day 2/3 rats [52]. Neurons, COS-7, and CHO-K1 cells were cultured and transfected as described earlier [23]. For indirect immunofluorescence analysis, hippocampal neurons, COS-7, and CHO-K1 cells were cultured on glass coverslips coated with either poly-D-lysine or poly-L-ornithine (P2/3 neurons) in 24-well plates. Hippocampal neurons were transfected at day-in-vitro 6–7 using Effectene transfection reagent (QIAGEN, Maryland, MD) with transfection efficiency of ~1%. After 2–3 h of incubation at 37°C, the transfection solution was replaced with a 50:50 solution of fresh/conditioned medium. COS-7 and CHO-K1 cells were transfected using Lipofectamine 2000 reagent (Life Technologies) for 5 h at 37°C with a transfection efficiency of ~60%.

### DNA constructs

As described in detail earlier, the C-terminal fusion proteins of EGFP and mouse, rat, or human GAD67 were generated by PCR using mouse, human, or rat GAD67 in pSV-SPORT as templates. The PCR products were subcloned into the HindIII and KpnI sites in-frame with EGFP in pEGFP-N3 (Clontech, Palo Alto, CA) [23]. The mouse, rat, and human GAD67-GFP proteins have been shown to share similar characteristics including membrane association and subcellular distribution in neurons [23]. The construction of the C-terminal fusion protein of EGFP and human GAD65 (hGAD65-GFP) was described previously [18]. The construction of the mammalian expression vector containing mCherry–tagged human GAD65 fusion protein (hGAD65-mCherry) was described previously [23].

### Immunofluorescence analyses

Immunofluorescence analysis of primary rat hippocampal neurons, COS-7, CHO, INS-1, and MIN6 cells was performed as described previously [23]. Mouse and rat islet single cells were fixed with 4% paraformaldehyde for 30 min and then permeabilized for 1 h with permeabilization buffer (PBS containing 0.5% Triton X-100 and 0.2% bovine serum albumin). Cells were blocked using animal-free blocker (Vector, Burlingame, CA) diluted 5-fold with water for 30 min and incubated with primary antibodies in permeabilization solution for 2 h. Cells were incubated with the respective secondary antibodies in permeabilization solution for 1 h with gentle shaking. To stain the nuclei, cells were incubated with the nuclear stain Hoechst 333432 (10 μg/ml in PBS) for 15 min. The coverslips were mounted in Prolong Gold antifade reagent (Life Technologies). The images were captured using a Leica TCS SP2 or SP5 laser scanning confocal fluorescence microscopes equipped with acousto-optical beam splitters and an HCX Plan-Apochromat 63x/1.20 NA oil objective. Images of 8-bit depth were collected using the Leica confocal software. All confocal images represent projections derived from 8–10 consecutive horizontal optical sections estimated at 0.5–1 μm in thickness. Final brightness/contrast picture adjustment and layout of the figures were done using Photoshop software (Adobe Systems, San Jose, CA).

A co-localization analysis of GAD67-GFP with GAD65-mCherry or the Golgi marker GM130 and of endogenous GAD67 with endogenous GAD65 or the Golgi marker GM130 was performed on projected confocal images using the correlation plot application in MetaMorph software (Universal Imaging, Sunnyvale, CA). A second set of co-localization analysis was performed on GAD65-GFP or endogenous GAD65 with the Golgi markers GM130 and giantin, or insulin. When measuring the co-localization with Golgi marker, the Golgi marker with red fluorescence was designated as the region of interest during the analysis and then this image was thresholded prior to the measurement, so that only pixels that have intensities that are outside of the threshold range were excluded from the measurement. When measuring the co-localization with cytosolic vesicles, the whole cell image was designated as the region of interest during the analysis. The calculation of the correlation coefficient by MetaMorph was described previously [12]. All data represented as mean ± SEM. To establish significance, results of quantification were analyzed by unpaired Student’s *t*-test using a two-tailed distribution and two-sample equal variance. The statistical analysis was performed using GraphPad Prism version 4.0 (GraphPad Software, San Diego, CA).

### Subcellular fractionation

Subcellular fractionation of INS-1 cells transiently expressing GAD67-GFP or GAD65-GFP, and mouse and rat islets was carried out as described previously [22]. Mouse islets were isolated from twenty 5-weeks-old C57BL/6 mice and rat islets were isolated from twenty 7-days-old Sprague Dawley rat pups. After 48 h of pre-culture, approximately 3000 islets were hand-picked again, washed once with 10 ml of ice-cold Ca^2+^/Mg^2+^-free Dulbecco’s phosphate buffered saline (DPBS) (Life Technologies), homogenized on ice for 20 min using a glass homogenizer, and subjected to subcellular fractionation as described above.

### Western blot analysis

Proteins were separated by SDS/PAGE on 10% NuPAGE Novex Bis-Tris mini gels (Life Technologies) and electroblotted onto nitrocellulose membranes. After the transfer, membranes were blocked with TBST (20 mM Tris/HCl, pH 7.5, 150 mM NaCl and 0.1% Tween 20) containing 10% non-fat dried skimmed milk powder for 1 h at room temperature. The filter membranes were then incubated for 1 h at room temperature with primary antibodies diluted in TBST containing 5% nonfat dry milk. The 1701 rabbit polyclonal antibody was used at 1:5,000 dilution, the mouse monoclonal antibody to GFP was used at 1:20,000 dilution, and the NAP22 antibody was used at 1:1000 dilution. Membranes were washed with TBST and then incubated for 1 h at room temperature with secondary horseradish peroxidase (HRP)-coupled sheep anti-mouse IgG or donkey anti-rabbit IgG (GE Healthcare, Buckinghamshire, UK) at 1:40,000 dilution in TBST containing 5% nonfat dry milk. The blots were developed using SuperSignal West Femto Maximum Sensitivity Chemiluminiscent Substrate (Thermo Scientific, Rockford, IL) and visualized on Hyperfilm ECL X-ray films (GE Healthcare). Scanning of autoradiograms was performed using EPSON Perfection 1200U scanner. For densitometry analysis, the band intensity of GAD65 and GAD67 were quantified by calculating the area under the curve of the specific signal using ImageJ software (NIH, Bethesda, MD). Immunostaining with the NAP22 antibody, was followed by incubation with an IRDye labeled anti-IgG secondary antibody (Odyssey, LI-COR, Lincoln, NE) at 1:15,000 dilution and imaged with an Odyssey Infrared Imager (LI-COR).

### Ethics Statement

Human islets for research were received from the University Hospital of Geneva thanks to the European Consortium for Islet Transplantation (ECIT) through islet distribution network (http://ecit.dri-sanraffaele.org/en/login/index.html) and analyzed at École Polytechnique Fédérale de Lausanne (EPFL). The use of human islet preparations for experimental research through ECIT was approved by the Institutional Review Board for clinical research of the Departments of Neurology, Dermatology, Anesthesiology and Surgery of the University Hospital of Geneva (CER Nr. 05–028). The University of Geneva Ethical Board waived the need for a consent from donors because islets were used for experimental research only when not suitable for clinical purposes and would otherwise have been destined for destruction. In such cases, obtaining informed consent is not mandatory in Switzerland. While EPFL does not have an institutional review board for research using human material, permit is granted for its use as long as the provider can certify that the samples were obtained according to local laws, regulations, and good practices. The study of rat and mouse islets and rat neurons was approved by the institutional review boards for the University of California San Francisco (UCSF), École Polytechnique Fédérale de Lausanne (EPFL), and University of Copenhagen. The numbers of the IACUC ethical approvals were for UCSF: AN083181 and AN091704, for EPFL: 2567, and for University of Copenhagen: 2008/561–1515.

## Results

### INS-1 and MIN6 insulinoma cells lack the GAD65 independent mechanism for membrane anchoring and targeting of GAD67 to membrane compartments

**Fig 1:**
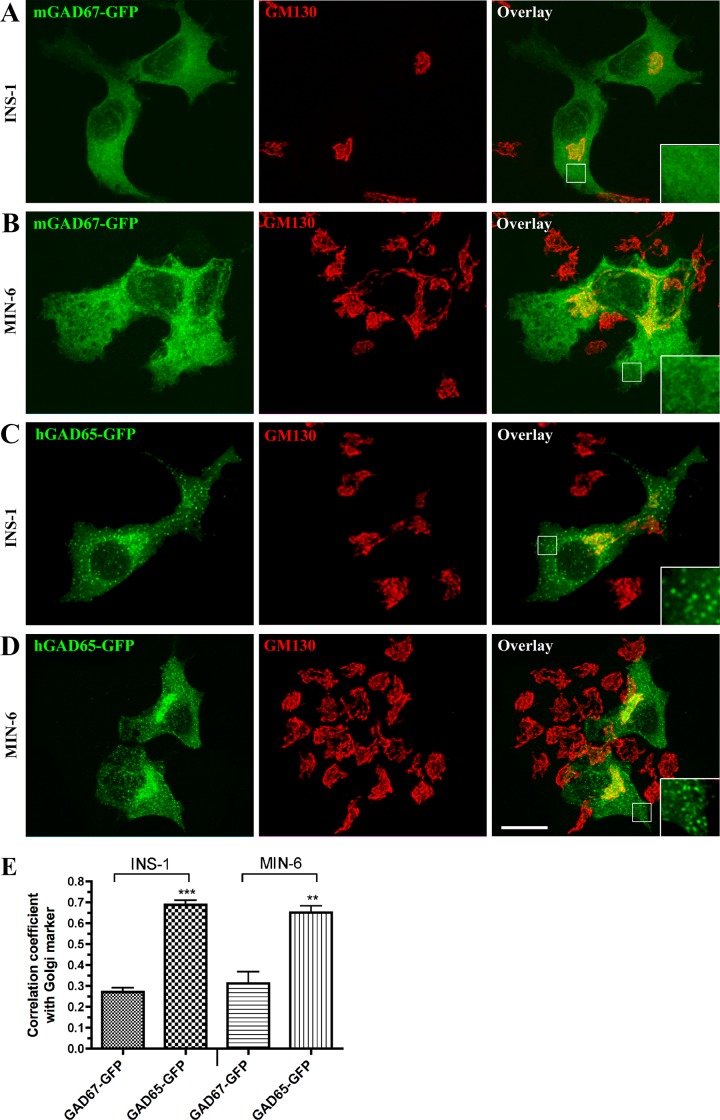
In the absence of GAD65, GAD67-GFP is cytosolic in INS-1 and MIN6 cells. Projected confocal images of INS-1 cells (**A**, **C**), and MIN6 cells (**B**, **D**) transiently expressing either mGAD67-GFP (**A**, **B**) or hGAD65-GFP (**C**, **D**). Cells were fixed 24 h following transfection and then immunostained for GFP (green) and the Golgi marker GM130 (red) before confocal analyses. GAD67-GFP is detected in an even diffuse pattern throughout the cytosol of INS-1 and MIN6 cells and does not appear to concentrate in the Golgi (red) or vesicular membrane compartments (A, B, enlarged frames). In contrast, hGAD65-GFP is detected in the Golgi compartment and in vesicular compartments in INS-1 and MIN6 cells (C, D; enlarged frames). Scale bar: 10 μm. (**E**) Correlation coefficient analysis for co-localization of GAD67-GFP and GAD65-GFP with GM130 in INS-1 and MIN6 cells. Results are presented as mean ± S.E. for 15 cells for each protein in INS-1 cells and for 6 cells for each protein in MIN6 cells. **P < 0.002; ***P < 0.0001

Transfected GAD67-GFP is efficiently targeted to Golgi membranes, somatic vesicles, and presynaptic nerve terminals in neurons independent of GAD65 [23] (S1 Fig.). We assessed whether GAD67-GFP and GAD65-GFP similarly acquire membrane anchoring in the insulinoma cell lines, INS-1 and MIN6, which lack the expression of endogenous GAD65 and GAD67 proteins. Confocal analyses of INS-1 and MIN6 cells, singly transfected with mGAD67-GFP revealed that, in contrast to neurons and several non-neuronal cell lines [23] (S1 Fig.), GAD67-GFP does not concentrate in the Golgi compartment or in cytosolic vesicles in the absence of GAD65, and is instead detected in a diffuse uniform pattern in the cytosol of insulinoma cells (Fig. 1A and B). In contrast, transfected hGAD65-GFP was concentrated in the Golgi compartment, where it colocalized with the Golgi marker GM130, and in cytosolic vesicles (Fig. 1C-E) distinct from insulin-containing secretory granules (S2 Fig.). These results suggest that, in contrast to neurons and some non-neuronal cell lines, the GAD65-independent membrane anchoring machinery is missing in INS-1 and MIN6 cells.

**Fig 2:**
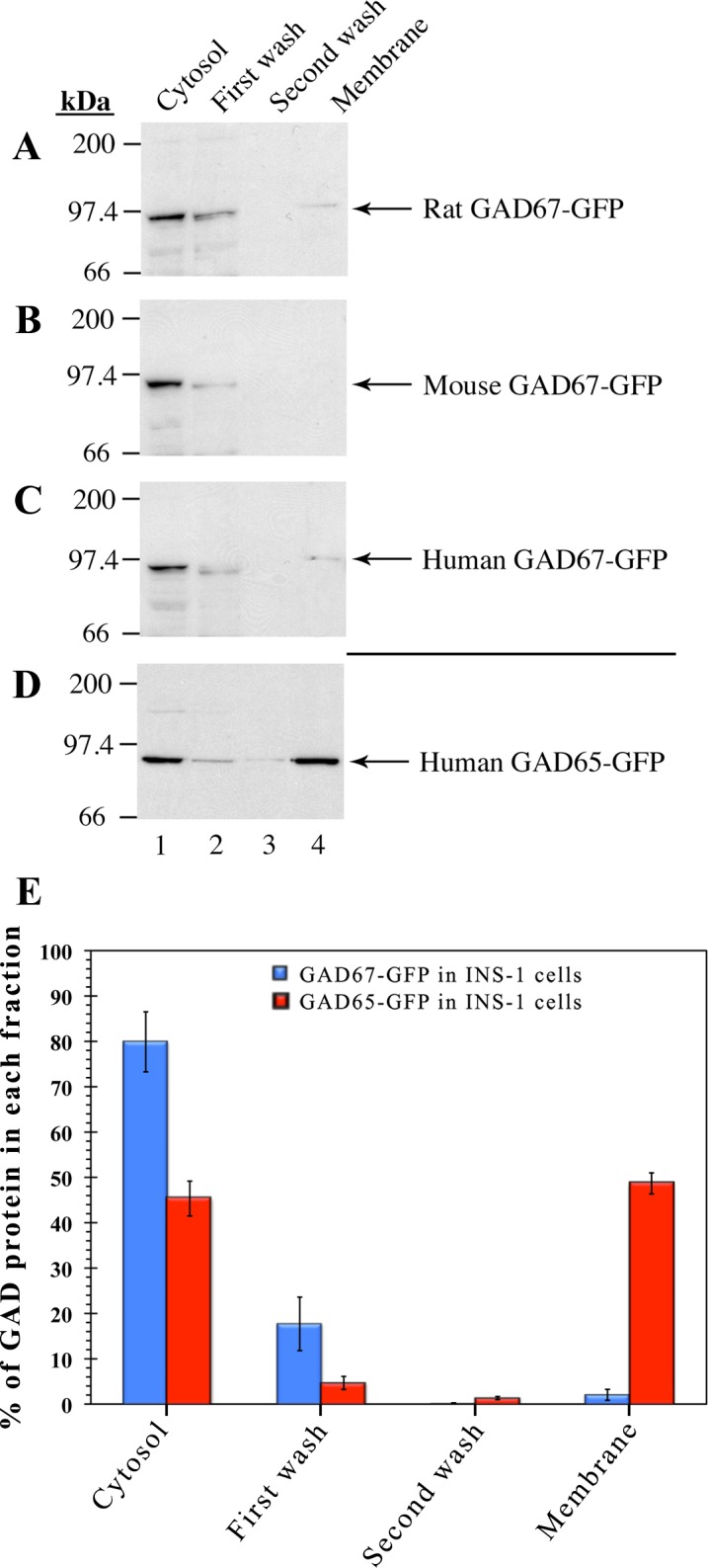
In the absence of GAD65, subcellular fractionation of INS-1 cells reveals lack of membrane targeting of GAD67-GFP. Immunoblotting analysis of the subcellular distribution of rat, mouse, and human GAD67-GFP in INS-1 cells (**A-C**), and human GAD65-GFP in INS-1 cells (**D**). INS-1 cells expressing either recombinant GAD67-GFP or GAD65-GFP proteins were subjected to subcellular fractionation using homogenization conditions that release most of the hydrophobic weakly membrane associated form of GAD65 into the soluble cytosolic fraction. Postnuclear supernatants were separated into a cytosolic fraction (lane 1), proteins washed from membranes by high salt (0.5 M NaCl, lanes 2 and 3), and firmly membrane anchored proteins extracted with 1% TX-114 (lane 4) were subjected to SDS-PAGE and immunoblotting with mouse anti-GFP antibodies. (**E**) Quantitative analysis of the subcellular distribution of GAD67-GFP and GAD65-GFP in INS-1 cells. Quantitation of GAD-GFP is expressed as the percentage of total GAD. Data are presented as mean ± S.E., n = 3. While GAD65-GFP expressed in INS-1 cells is equally distributed between the cytosol (45.3 ± 3.8%) and firmly membrane bound (48.7 ± 2.3%) fractions, most of GAD67-GFP in INS-1 cells is distributed in the cytosol (79.9 ± 6.6%) and first membrane wash (17.7 ± 5.9%). Thus, while human GAD65-GFP is equally distributed between the cytosol fraction and in the washed membrane fraction, GAD67-GFP transiently expressed in INS-cells is primarily detected in the cytosol and the first membrane wash fractions in the absence of GAD65.

Subcellular fractionation analyses of brains from GAD65^-/-^ mice have shown that about half of endogenous neuronal GAD67 is firmly membrane anchored in the absence of GAD65 [22]. Similar results were obtained in COS-7 cells singly transfected with mouse or human GAD67 [23]. These results are consistent with confocal analysis showing targeting of GAD67-GFP to membrane compartments in neurons and several cell lines in the absence of GAD65 [23] (S1 Fig.). While the confocal analyses of GAD67-GFP in INS-1 and MIN6 cell lines showed a uniform cytosolic pattern, it did not exclude that a fraction of the protein was anchored to Golgi membranes (Fig. 1, panels A, B, overlay). To assess whether a fraction of GAD67-GFP was membrane anchored in these cells, INS-1 cells singly transfected with rat, mouse, or human GAD67-GFP, were subjected to subcellular fractionation. The results were compared to the subcellular distribution of singly transfected hGAD65 (Fig. 2). We used a hypotonic homogenization buffer containing 1mM MgCl_2_, which releases most of the hydrophobic peripheral weakly membrane associated form of GAD65 into the cytosolic fraction [17]. Membranes were washed twice in a high salt solution (0.5 M NaCl), first to remove traces of cytosolic proteins, and then any remaining peripheral membrane-associated proteins, before extraction of firmly membrane-anchored proteins with detergent [16, 17]. Analysis of post-nuclear subcellular fractions (Fig. 2) revealed that singly transfected GAD67-GFP in INS-1 cells is primarily detected in the cytosolic and first membrane wash fractions (Fig. 2A-C, E). In contrast, about half of singly transfected hGAD65-GFP in INS-1 cells was detected in washed membrane fractions (Fig. 2D and E). These experiments provide additional evidence that INS-1 cells lack the GAD65-independent membrane anchoring mechanism of GAD67.

### Endogenous GAD65 and transfected GAD65-GFP target to Golgi and vesicular membrane compartments in primary β-cells

**Fig 3:**
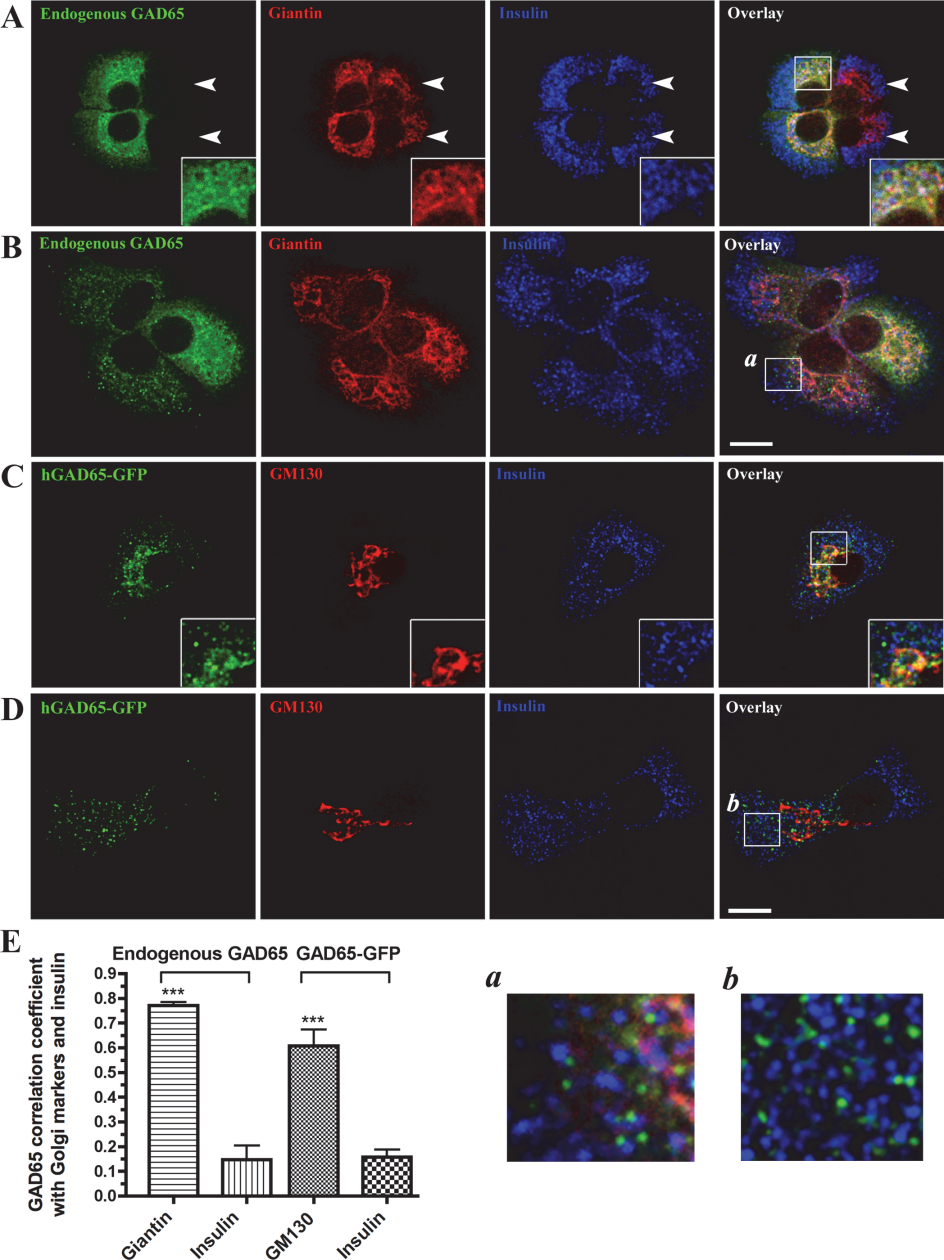
Endogenous GAD65 and transfected GAD65-GFP in rat pancreatic β-cells are primarily targeted to Golgi and vesicular membrane compartments. Projected confocal images of endogenous GAD65 (**A**, **B**) and transfected hGAD65-GFP (**C, D**) in rat islet single cells. Cells in panels A and B were immunostained for endogenous GAD65 (GAD6 antibody, green), the Golgi marker giantin (red), and insulin (blue). Cells in panels C and D were fixed 48 hours following transfection and immunostained for GFP (green), the Golgi marker GM130 (red), and insulin (blue). Endogenous GAD65 and transfected hGAD65-GFP are expressed in the Golgi compartment of rat pancreatic β-cells (A and C, enlarged frames), and in cytosolic vesicles that are distinct from the insulin-containing vesicles (B and D, enlarged frames). Scale bar: 10 μm. (**E**) Correlation coefficient analysis for co-localization of endogenous GAD65 or transfected hGAD65-GFP with either giantin/GM130 or insulin. Results are presented as mean ± S.E. *n* = 6–24 cells per group. ***P < 0.0001.

INS-1 and MIN6 cells have lost the expression of endogenous GAD65 and GAD67, and may also have lost expression of components required for aspects of membrane anchoring and trafficking of the GAD isoforms. To study the subcellular localization of GAD65 and GAD67 in primary β-cells, we developed monolayer cultures of dispersed islet cells prepared from rat, mouse, or human islets and grown on cover slips. We first analyzed the distribution of endogenous GAD65 in monolayer cultures of rat islets cells, immunostained for GAD65, the Golgi marker protein giantin, and insulin. Fig. 3, panels A and B show representative results of three different independent experiments in rat islet cells. Endogenous GAD65 was expressed in both the Golgi compartment and in vesicles distinct from insulin containing vesicles (Fig. 3A, B, E) as shown in INS-1 cells (S2 Fig.). A similar pattern of expression of endogenous GAD65 was also observed in monolayers of human islets (S3 Fig.) and in intact rat and human islets in culture. We observed that in the cultures of neonatal rat islet cells, a fraction (approximately 20%) of cells positive for insulin staining did not express endogenous GAD65 at detectable levels (see cells indicated with arrow heads in Fig. 3A and S4 Fig.). This is in contrast to the situation in adult rat islets [28] and adult human islet cells where every insulin positive cell also expresses GAD65 and suggests that insulin expression may precede GAD65 expression in newly differentiated β-cells in neonatal rat islets. We were unable to detect GAD65 expression in any insulin negative cells including glucagon positive α-cells (S4 Fig.). A similar pattern of expression was observed when single cells from neonatal rat islets were transfected with hGAD65-GFP. Fig. 3, panels C, D, E show representative results of four independent experiments. Notably, hGAD65-GFP expresses either entirely in the Golgi compartment in some cells (not shown), in both Golgi and cytosolic vesicles (Fig. 3C), or entirely in vesicles (Fig. 3D). The variations from cell to cell in the ratio between Golgi versus cytosolic expression of GAD65-GFP were consistent with observations in INS-1 and MIN6 cells (Fig. 1) and of endogenous GAD65 expression in rat and human islet cells (Fig. 3B and results not shown).

### Singly expressed endogenous GAD67 in islet β-cells is cytosolic, while GAD67 in β-cells expressing GAD65 is targeted to Golgi and vesicular membrane compartments

**Fig 4:**
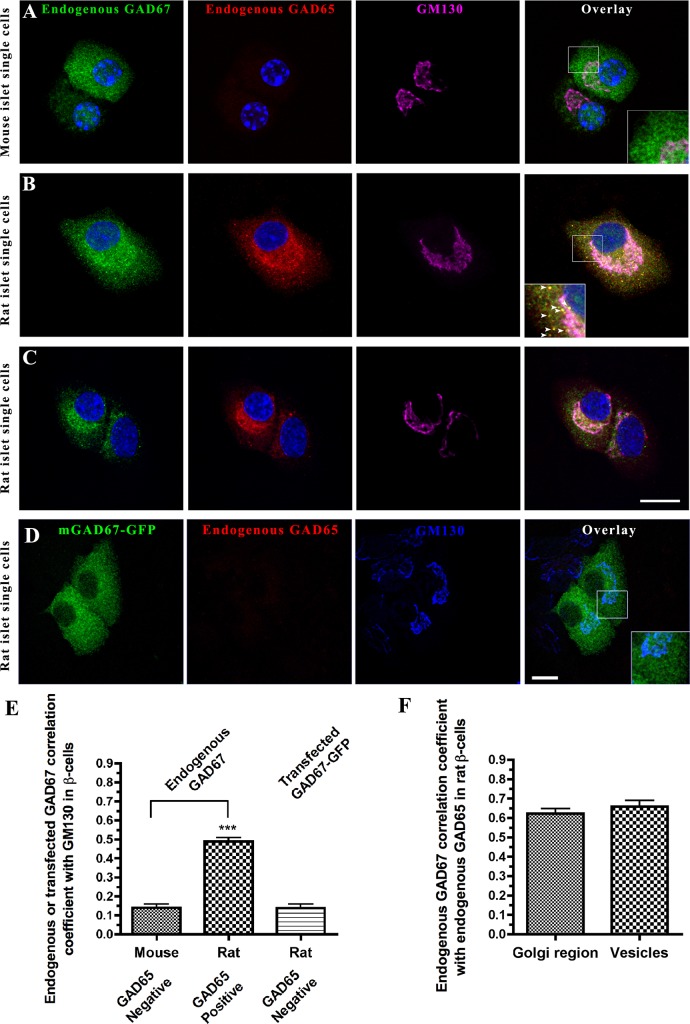
Confocal analyses of endogenous GAD67 and GAD65 in mouse and rat islet single cells reveal that GAD67 is cytosolic in the absence of GAD65 but targets to Golgi and vesicular membranes in the presence of GAD65. Projected confocal images of mouse **(A)** and rat **(B, C, D)** islet single cells immunostained for endogenous GAD67 (green, K2 rabbit polyclonal antibody), or transfected GAD67-GFP (green, antibody to GFP) and endogenous GAD65 (red, human monoclonal antibodies MICA 2, 3, and 6), the Golgi marker protein GM130 (purple), and the nuclear stain Hoechst 333432 (blue). (**A**) Mouse islet single cells express the GAD67 isoform and lack the expression of detectable levels of endogenous GAD65. In these GAD65-deficient cells, endogenous mouse GAD67 is detected as a cytosolic protein and is absent from Golgi membranes and cytosolic vesicles (A, enlarged frame). Thus, mouse islet β-cells lack the GAD65-independent machinery for trafficking GAD67 through membrane compartments to reach peripheral vesicles. (**B, C**) Rat islet β-cells, however, express both the endogenous GAD67 and GAD65 isoforms. In these cells, GAD67 and GAD65 are expressed similarly in Golgi membranes and cytosolic vesicles (B, arrowheads in enlarged frame). (**D**) In the subpopulation of rat islet single cells that are devoid of endogenous GAD65, transfected mGAD67-GFP shows a cytosolic distribution and fails to associate with Golgi membranes and cytosolic vesicles (D, enlarged frame). Scale bar: 10 μm. (**E**) Correlation coefficient analysis for co-localization of endogenous GAD67 or transfected GAD67-GFP with GM130 in mouse and rat islet single cells. (**F**) Correlation coefficient analysis for the co-localization of endogenous GAD67 with endogenous GAD65 in the Golgi region and cytosolic vesicles in rat islet single cells. Results are presented as mean ± S.E. *n* = 5–15 cells per group. ***P < 0.0001.

To address the possibility that primary β-cells have preserved the GAD65 independent mechanism of membrane anchoring of GAD67, we exploited the intrinsic differences in GAD65 expression between mouse and rat islet β-cells, comparatively analyzing the expression of endogenous GAD67 in primary cultures of mouse islet cells that lack detectable expression of GAD65 and in rat islet cells that express both isoforms of GAD [25, 53]. As expected, high-resolution confocal analysis revealed no signal for endogenous GAD65 in any of the mouse islet β-cells (Fig. 4A). In these GAD65-deficient cells, endogenous mouse GAD67 was absent from Golgi membranes and cytosolic vesicles and instead appeared evenly distributed in the cytosol (Fig. 4A, E). In contrast, in rat islet single cells, endogenous GAD67 and GAD65 co-localized in Golgi membranes (Fig. 4B-F) and in vesicular compartments (Fig. 4B, enlarged frame, 4F). Finally, to study the targeting of GAD67-GFP in the absence of GAD65 in primary rat islet cells, neonatal rat islet cells were singly transfected with mGAD67-GFP followed by immunostaining of fixed cells for GFP, endogenous GAD65, and the Golgi marker protein GM130. We identified transfected islet cells that were negative for endogenous GAD65, and are therefore either GAD65-negative beta cells, such as those shown in Fig. 3A, or islet non-β-cells. mGAD67-GFP in GAD65 negative rat islet cells was not targeted to Golgi membranes or cytosolic vesicles, and instead was evenly distributed in the cytosol (Fig. 4D, E). Thus GAD67 in islet cells appears to only target to membrane compartments when co-expressed with GAD65.

**Fig 5:**
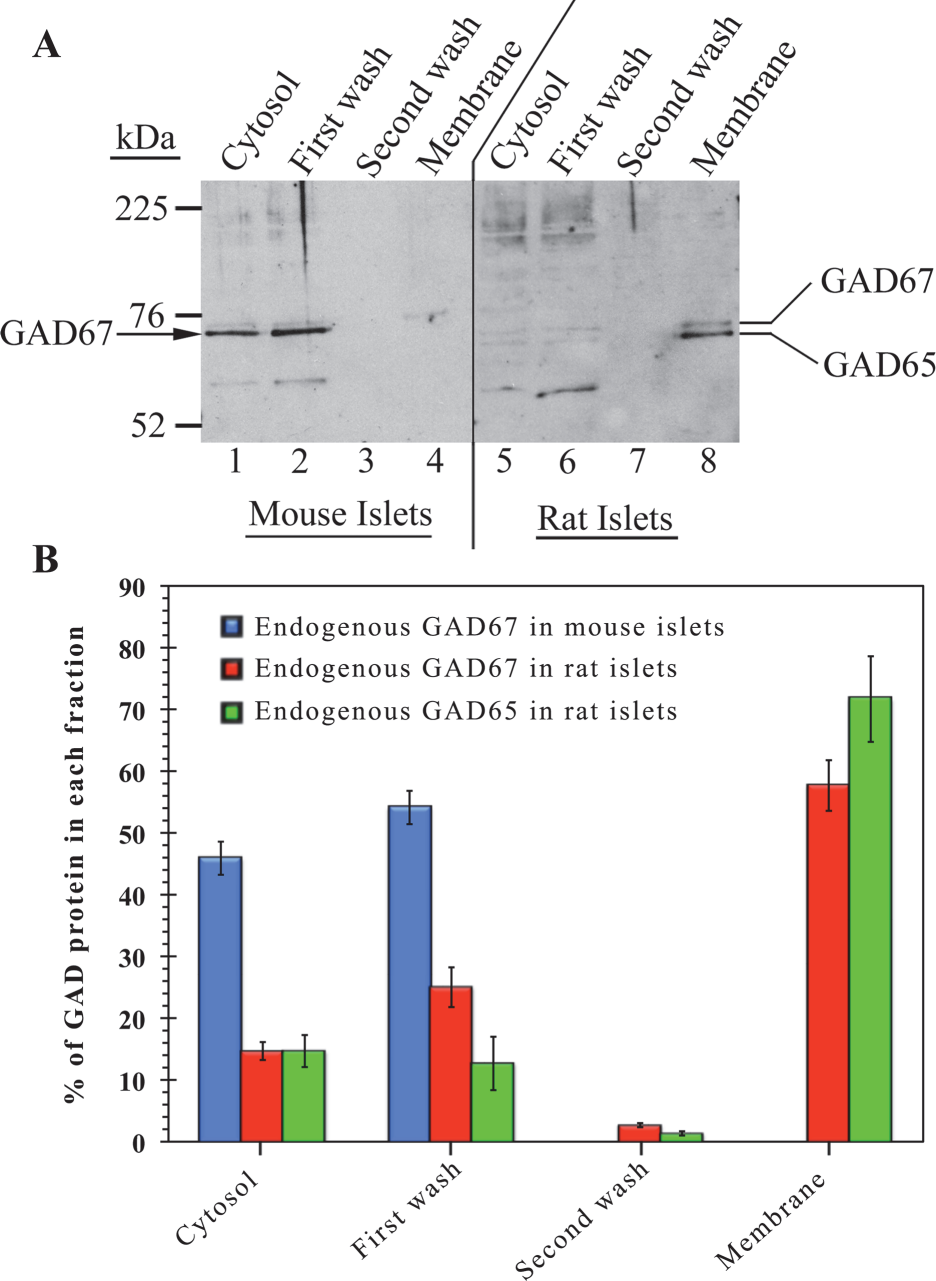
Subcellular fractionation of mouse and rat islets reveals lack of membrane anchoring of endogenous GAD67 in the absence of GAD65. (**A**) Western-blot analysis of the subcellular distribution of endogenous GAD67 in the absence of endogenous GAD65 in mouse islets (lanes 1–4) as compared to its distribution in the presence of GAD65 in rat islets (lanes 5–8). Mouse and rat islets were subjected to subcellular fractionation. Cytosolic proteins (lanes 1 and 5), proteins washed from membranes by high salt (lanes 2–3 and 6–7), and detergent extracted membrane proteins (lanes 4 and 8) were subjected to SDS-PAGE and immunoblotting with the 1701 antibody, which recognizes both GAD65 and GAD67. (**B**) Quantitative analysis of the subcellular distribution of endogenous GAD67 and GAD65 in mouse and rat islets. Quantitation of the immunoblots was performed using NIH ImageJ software. GAD immunoreactivity in each fraction is expressed as the percentage of total GAD. Data are presented as mean ± S.E. (*n* = 3). In the absence of GAD65 in mouse islets, GAD67 is detected similarly in the cytosol (45.9 ± 2.7%) and first membrane wash (54.1 ± 2.7%) and is completely absent in the membrane fraction. However, in the presence of GAD65 in rat islets, a large percentage of GAD67 (57.7 ± 4.0%) is detected together with GAD65 (71.7 ± 6.9%) in the washed membrane fraction.

As shown in Figs. 1 and 2, singly transfected GAD67-GFP was detected in a diffuse cytosolic pattern and >90% is localized in the cytosol fraction of the insulinoma cell lines INS-1 and MIN6. However, the appearance of endogenous GAD67 in mouse islet cells (Fig. 4A) was less diffuse in appearance in confocal images than GAD67-GFP in insulinoma cells (Fig. 1A, B). To further study the subcellular localization of endogenous GAD67 in the absence and presence of endogenous GAD65, mouse and rat islets were subjected to subcellular fractionation analysis followed by SDS-PAGE and Western blotting with the 1701 rabbit polyclonal antibody that recognizes GAD65 and GAD67 with similar affinity. Subcellular distribution analysis revealed that in the absence of GAD65 in mouse islets, GAD67 is about equally distributed between the cytosol and first membrane wash fractions (Fig. 5A, lanes 1 and 2), and is completely absent from the firmly membrane-anchored protein fraction extracted from washed membranes by detergent (Fig. 5, lane 4). However, in rat islets, the majority of GAD67 is detected together with GAD65 in the firmly membrane anchored protein fraction (Fig. 5A, lane 8). Taken together, the results provide evidence that mouse β-cells lack the GAD65-independent machinery for membrane anchoring and trafficking of GAD67 through membrane compartments to reach peripheral vesicles. However, the increased amount of GAD67 distributed into the first membrane wash as well as the confocal appearance of the protein in an extensive cytosolic network suggest that mouse islet cells may differ from insulinoma cells in having the ability to mediate weak reversible peripheral membrane association of GAD67. The results also suggest that the presence of GAD65 in rat islet cells results in membrane anchoring and targeting of GAD67 to the Golgi compartment and vesicular compartments. Thus, similar to the situation in primary rat hippocampal neurons [22, 23], we propose that GAD67 in β-cells piggy-backs onto the membrane anchoring and trafficking pathway of GAD65 through heterodimerization.

### In primary pancreatic β-cells and in insulinoma cells, transiently expressed GAD67-GFP traffics to Golgi membranes and vesicular compartments via its association with GAD65-mCherry

**Fig 6:**
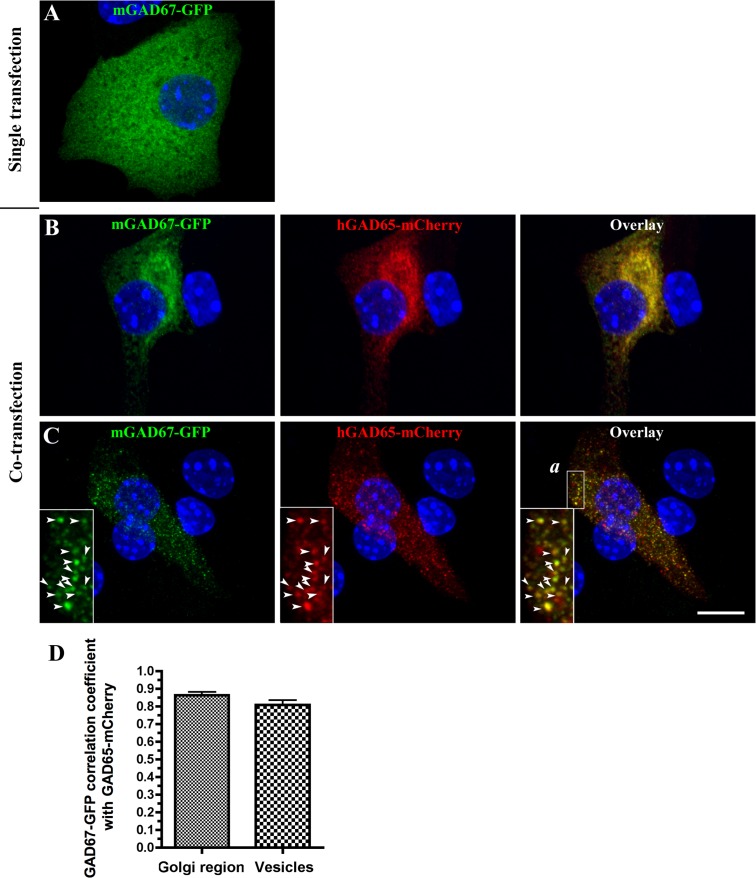
Targeting of mGAD67-GFP to Golgi and vesicular membrane compartments in GAD65 deficient mouse islet cells by co-expression with hGAD65-mCherry. Projected confocal images of mouse islet cells singly transfected with mGAD67-GFP (**A**) or doubly transfected with mGAD67-GFP and hGAD65-mCherry (**B**, **C**). Cells were fixed 48 h following transfection and immunostained for GFP (green) and the nuclear stain Hoechst 333432 (blue) (A, B, C) and mCherry (red) (B, C). Singly transfected mGAD67-GFP shows a cytosolic distribution and does not associate with Golgi membranes and cytosolic vesicles. However, co-expression with hGAD65-mCherry results in the targeting of mGAD67-GFP to Golgi membranes (B) and cytosolic vesicles (C, enlarged frame *a*) where it colocalizes with hGAD65-mCherry. Scale bar: 10 μm. (**D**) Correlation coefficient analysis for co-localization of mGAD67-GFP with hGAD65-mCherry in the Golgi region and cytosolic vesicles of doubly transfected mouse islet single cells. Results are presented as mean ± S.E. for 9 cells analyzed for co-localization in Golgi region and 12 cells analyzed for co-localization in cytosolic vesicles.

The results described above do not directly show that expression of GAD65 in rat islet cells mediates targeting of GAD67 to membrane compartments. To further assess the role of GAD65 expression on GAD67 membrane anchoring and targeting, we transfected GAD67-GFP into primary mouse and rat islet cells and insulinoma cells either singly or together with GAD65-mCherry. The mCherry construct encodes a protein that has previously been shown to target to Golgi membranes, cytosolic vesicles, and presynaptic nerve terminals in rat hippocampal neurons, similarly to endogenous GAD65 [23]. The subcellular localization of transfected GAD65-mCherry and GAD67-GFP in islet and insulinoma cell cultures was studied using high-resolution confocal analyses. While singly transfected hGAD65-mCherry was targeted to Golgi membranes and peripheral vesicles in mouse islet cells (results not shown), singly transfected mGAD67-GFP was absent from Golgi membranes and vesicular compartments and instead distributed evenly in a cytosolic network (Fig. 6A) similar to the expression of endogenous GAD67 in mouse islet cells (Fig. 4A). In contrast, when mGAD67-GFP was co-expressed with hGAD65-mCherry in mouse islet-derived single cells, it was targeted to Golgi membranes (Fig. 6B) and vesicular compartments (Fig. 6C, enlarged frame *a*), where it colocalized with hGAD65-mCherry.

**Fig 7:**
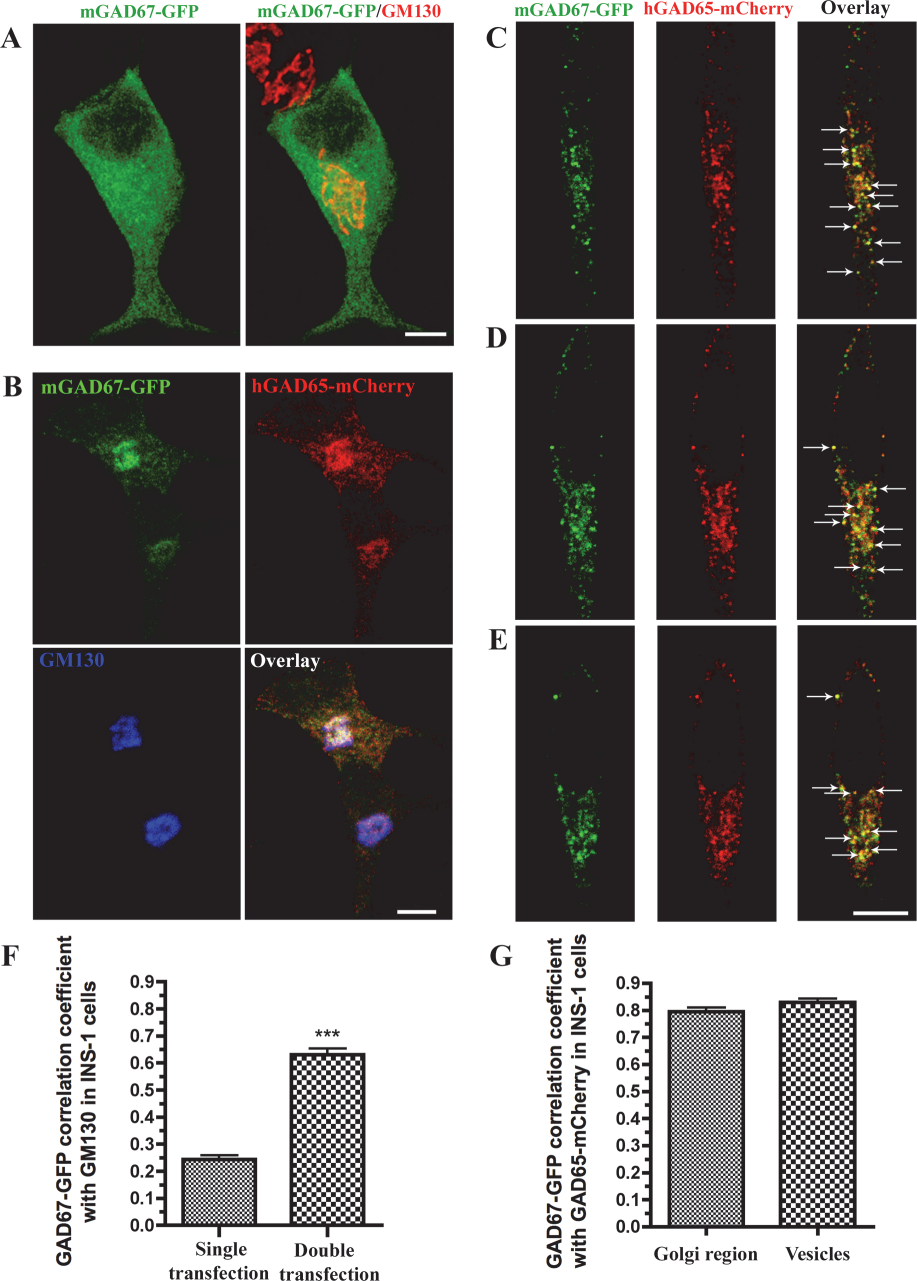
Human GAD65-mCherry targets mouse GAD67-GFP to cytosolic vesicles and Golgi membranes in INS-1 cells. **(A)** Projected confocal images of INS-1 cell singly transfected with mGAD67-GFP and immunostained for GFP (green) and the Golgi marker GM130 (red). **(B)** Projected confocal images of INS-1 cells transfected with mGAD67-GFP and hGAD65-mCherry and triple immunostained for GFP, mCherry, and GM130. **(C-E)** selected confocal stacks (~0.5 μm in thickness) of INS-1 cells co-expressing mGAD67-GFP and hGAD65-mCherry and immunostained for GFP and mCherry. Co-expression with hGAD65-mCherry results in targeting of cytosolic mGAD67-GFP (A) to Golgi membranes where it co-localizes with hGAD65-mCherry and GM130 (white color, B) and to cytosolic vesicles that also harbor hGAD65-mCherry (orange color, indicated by arrows, C-E). Scale bars: 20 μm. (**F**) Correlation coefficient analysis for co-localization of mGAD67-GFP with GM130 in singly (n = 12) and doubly (n = 7) transfected INS-1 cells. (**G**) Correlation coefficient analysis for co-localization of mGAD67-GFP and hGAD65-mCherry in Golgi region (n = 11) and cytosolic vesicles of doubly transfected INS-1 cells (n = 23). Results are presented as mean ± S.E. ***P < 0.0001.

Finally, we addressed the question whether GAD65-mCherry can target cytosolic mGAD67-GFP to Golgi membranes and cytosolic vesicles in INS-1 cells. High-resolution confocal analyses revealed that, in contrast to the cytosolic distribution of singly transfected mGAD67-GFP in INS-1 cells (Fig. 7A), co-transfection with hGAD65-mCherry resulted in targeting of mGAD67-GFP to Golgi membranes, where it co-localized with hGAD65-mCherry and the Golgi marker protein GM130 (Fig. 7B, F, G) and to cytosolic vesicles that also harbor hGAD65-mCherry (confocal stacks, Fig. 7C-G). The results in transfected primary mouse β-cells, rat islet cells, and insulinoma cells, collectively provide strong evidence that the GAD65-independent mechanism of GAD67 membrane anchoring is lacking in islet and insulinoma cells. Despite differing in their expression of GAD65, which resulted in Golgi trafficking of GAD67 by piggybacking with GAD65 in rat but not mouse islets, neither rat or mouse islets evidenced a GAD65-independent mechanism for GAD67 transport. Furthermore, GAD65 expression is sufficient to mediate membrane anchoring of GAD67 and targeting to Golgi membranes and cytosolic vesicles in both mouse and rat β-cells. Thus, the GAD65-dependent mechanism of GAD67 membrane anchoring is shared between neurons and β-cells, whereas the GAD65-independent mechanism for GAD67 is lacking in the insulin-producing islet cells.

Both GAD67 and GAD65 were amongst several proteins identified in a pull-down assay of rat brain extracts using a neuron enriched lipid raft protein NAP22 as a bait. Based on this result, the NAP22 protein was suggested to play a role in targeting of the GAD isoforms to membranes [57]. While the membrane anchoring of GAD65 is similar in β-cells, INS-1 cells, and neurons and seems to be independent of association with other proteins, as shown here and in other studies [12, 13, 16–19, 21], membrane anchoring mechanism for the hydrophilic GAD67 protein in the absence of GAD65 appears to involve association with a distinct hydrophobic membrane protein or membrane anchoring complex [23]. We addressed the question whether the lack of the GAD65 independent membrane anchoring mechanisms for GAD67 in β-cells is associated with a lack of NAP22 expression in islet β-cells and INS-1 cells. Immunostaining of Western blots of islets and INS-1 cells, however, revealed that NAP22 is expressed in both cell types, albeit at lower levels than in brain extracts (S5 Fig.). Confocal analyses of expression and subcellular distribution of NAP22 in INS-1 cells and primary islet cells confirmed that NAP22 is expressed in insulinoma cells and in islet β- as well as non β-cells. In those cells, NAP22 is primarily localized in the plasma membrane where GAD65 and GAD67 are absent (S6A–B Fig.). There was no clear colocalization between singly transfected GAD67-GFP and NAP22 in INS1 cells or between endogenous GAD65 and NAP22 in primary rat islet cells. In neurons, colocalization of GAD65 with NAP22 was only detected in a small fraction of axonal clusters (S6C Fig.). These results suggest that the lack of GAD67 membrane association in β cells cannot be explained by a lack of NAP22 expression and that a possible association between NAP22 and the GAD isoforms is either rare or non-existent in β cells. In sum, a critical role of protein(s) distinct from NAP22 is inferred in membrane anchoring of GAD67.

## Discussion

We have previously shown in neurons, that the hydrophilic GAD67 isoform is anchored to membranes and robustly targeted to the Golgi compartment and presynaptic clusters either by association with the hydrophobic GAD65 isoform [22, 54] or by a distinct GAD65-independent mechanism that involves association with a different membrane-anchoring moiety [23].

In the current study, we have analyzed the membrane anchoring and subcellular destination of the two isoforms of GAD in pancreatic islet β-cells. The results presented here confirm that the smaller isoform, GAD65, is targeted to the Golgi compartment and peripheral vesicles in islet and insulinoma cells, similarly to the situation in neurons. In marked contrast, the larger isoform–GAD67–is completely dependent on GAD65 for targeting to membrane compartments in islet β-cells. As such, β-cells lack the GAD65-independent membrane anchoring mechanism for GAD67, which was identified in neurons and some non-neuronal cell lines [23]. Endogenous GAD67 is targeted to the Golgi compartment and peripheral secretory vesicles and is firmly membrane anchored in the presence of endogenous GAD65 in rat β-cells. Moreover, GAD65-mCherry targets GAD67-GFP to Golgi membranes and cytosolic vesicles in insulinoma cells and GAD65-deficient mouse β-cells or rat islet cells. Therefore, the GAD65-dependent, but not the GAD65-independent mechanism for targeting of GAD67 to membrane trafficking pathways, is functional in β-cells.

Notably, and in agreement with the ground-breaking studies by Sorenson et al., [28], GAD65- and GAD65/GAD67-positive vesicles in β-cells are distinct from insulin-containing vesicles and do not colocalize with the hormone. This result is consistent with an earlier immuno-EM study showing localization of GAD65 in Golgi membranes and membranes of small vesicles in β-cells but not in the large dense core vesicles (LDCV) that contain insulin [17]. However, GABA, the product of GAD65/67, has been detected in LDCVs in β-cells, suggesting that GABA released by GADs into the cytosol of β-cells can be incorporated into LDCVs [7, 34]. Furthermore, the detection of GABA in pancreatic islet cells may indicate that GABA released into the islet interstitium by β cells can be taken up by α-cells, perhaps by a reverse action of the plasma membrane GABA transporter GAT3 expressed in these cells [34].

**Fig 8:**
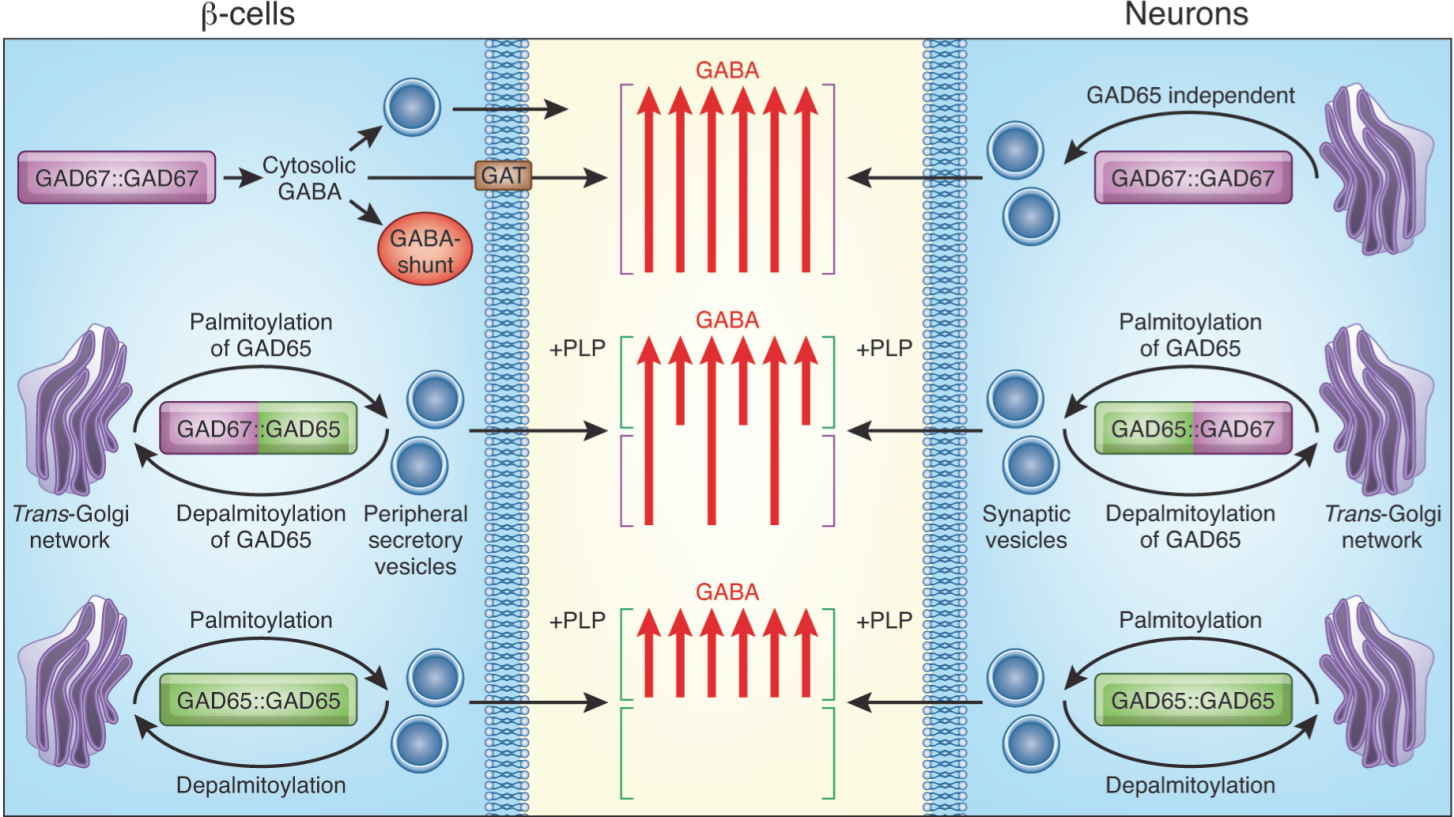
Schematic model of synthesis by and trafficking of GAD-dimers in β-cells and neurons. Three different dimers synthesize GABA in β-cells and neurons. Homodimers of GAD65, transiently activated by influx of PLP, traffic between the cytosolic face of TGN and peripheral vesicles in β-cells and synaptic vesicles in neurons. The anterograde trafficking from TGN is dependent on palmitoylation of cysteines 30 and 45 [19]. Depalmitoylation of the protein results in axonal retrograde transport back to the Golgi compartment [12]. Newly synthesized GABA produced by this dimer, may deliver GAD65 directly onto a vesicular GABA transporter for rapid accumulation and secretion. The GAD67:GAD65 heterodimer, consisting of one constitutively active GAD67 subunit and one conditionally active GAD65 subunit transiently activated by an influx of PLP, can be targeted to TGN and peripheral vesicles/presynaptic clusters through the membrane anchoring moiety of GAD65 [22] and perhaps also cycle between the TGN and peripheral vesicles via the palmitoylation/depalmitoylation cycle of GAD65. It is unknown whether GABA produced by GAD67 in this heterodimer, can be directly loaded onto a vesicular GABA transporter associated with GAD65, or whether it is first released into the cytosol. In neurons, the constitutively active GAD67:GAD67 homodimers, are anchored to membranes and target to Golgi and synaptic vesicle membranes by a GAD65 independent mechanism [23]. In β-cells, however, the GAD67 homodimers are, however, soluble and produce GABA released into the cytosol. Three possible fates can be suggested for GABA produced by this dimer. It could be secreted by a reverse orientation of the plasma membrane GABA transporter GAT3, transported to and taken up by secretory vesicles by an unknown mechanism, or metabolized in the GABA-shunt.

The native forms of the GAD enzymes involve non-disulfide linked dimers [8, 9, 55]. GAD forms three distinct dimers: a GAD65:GAD65 homodimer, a GAD67:GAD65 heterodimer, and a GAD67:GAD67 homodimer (Fig. 8). In neurons, all three dimers become targeted to Golgi membranes, to an axonal vesicular pathway, and to presynaptic clusters [18, 23]. In addition, axonal polarization and presynaptic clustering of all three dimers are mediated by the Rab5a-dependent pathway [24]. In pancreatic islet β-cells, however, only the GAD65:GAD65 homodimer and the GAD67:GAD65 heterodimer become targeted to Golgi membranes and cytosolic vesicles, while the GAD67:GAD67 homodimer is predominantly a soluble cytosolic protein. Each dimer has unique properties in terms of regulation of enzymatic activity and subcellular distribution. Thus, the GAD65 homodimer is either in the apo- or holo-enzyme form, depending on PLP levels, and serves to provide a dynamic enzyme reservoir that can rapidly be mobilized from the Golgi compartment for trafficking to presynaptic vesicles in axon termini in neurons and to peripheral secretory vesicles in β-cells, via the palmitoylation/depalmitoylation cycle of GAD65 [12]. The association of this dimer with a vesicular GABA transporter (VGAT) on the cytosolic face of synaptic vesicle membranes in neurons [56] and either VGAT or a distinct vesicular GABA transporter in β-cells [36] may facilitate a rapid vesicular accumulation of GABA.

The GAD65:GAD67 heterodimer combines the property of a constitutively active GAD67 monomer with the inducible activity of the GAD65 monomer, likely following the trafficking pathway of the GAD65 subunit, thus gaining access to the dynamic GAD65 palmitoylation/depalmitoylation cycle. In neurons, the GAD67:GAD67 homodimer is a constitutively active holoenzyme, capable of GABA synthesis not only in the cytosol but also in Golgi and vesicular membranes, thereby providing the main supply of basal GABA levels for regulated secretion in the course of inhibitory neurotransmission. In β-cells, however, the lack of flexible localization of the GAD67:GAD67 homodimer suggests that it produces only cytosolic GABA. We propose that cytosolic GABA produced by GAD67:GAD67 in β-cells can have multiple destinations and functions, including: i) release to the islet interstitium by a non-vesicular mechanism to produce tonic rather than phasic signaling through highly sensitive GABA_A_ receptors reported in islets [58]; ii) transport to and uptake by secretory vesicles for regulated secretion; iii) serve as a β-cell growth factor [31] perhaps secreted by a reverse action of GATs as described for neurons [59]; and iv) providing a source of energy through metabolizing in the GABA shunt [28]. Because of the species differences in β-cell GAD-isoform expression [25], it would appear that mouse islets only express the GAD67 homodimer, human islets only the GAD65 homodimer, while rat islets express all three dimers. Future studies of GABA secretion and function in islets of mouse, human and rat, may address the possibility that each GAD-isoform and each GAD-dimer is involved preferentially in a specific mode of GABA release and/or function in islet cells, and, therefore, that there may be subtle but important physiological differences in the roles of GABA biosynthesis in islet β-cells of the three species.

## Supporting Information

S1 Fig The GAD65-independent mechanism of GAD67 membrane anchoring is functional in COS-7 cells, CHO cells, and rat hippocampal neurons. Projected confocal images of a COS-7 cell (**A)**, CHO cell **(B**), and rat hippocampal neurons (**C-E**) transiently expressing GAD67-GFP. COS-7 and CHO cells were fixed 24 h following transfection. Rat hippocampal neurons were transfected at DIV 6 and fixed 72 h following transfection. (A-C) Cells were immunostained for GFP (green) and the Golgi marker GM130 (red). GAD67-GFP is targeted to Golgi membranes and cytosolic vesicles (A-C, enlarged frames) in COS-7 cells (A), CHO cells (B), and rat hippocampal neurons (C). (D-E) Rat hippocampal neurons were triple immunostained for GFP (green), synaptophysin (red), and endogenous GAD65 (GAD6 antibody, blue). The transfected neuron is a non-GABAergic neuron devoid of endogenous GAD65. However, GAD65 is seen in the cell body and axonal puncta of a neighboring GABAergic neuron (blue). Human GAD67-GFP colocalizes with synaptophysin in presynaptic clusters (arrowheads, enlarged frame), which are devoid of GAD65. Endogenous GAD65 colocalizes with synaptophysin in presynaptic clusters of the non-transfected GABAergic neuron in the same field of view (arrows, enlarged frame). Scale bars: 10 μm.
(TIF)

S2 Fig GAD65-GFP-containing vesicles in INS-1 cells are distinct from the insulin-containing large dense core secretory vesicles. Projected confocal images of INS-1 cells singly transfected with hGAD65-GFP and immunostained for GFP (green), endogenous insulin (red) and the nuclear stain DAPI (blue). GAD65-GFP-containing vesicles do not co-localize with insulin-containing large dense core vesicles (enlarged frame). Scale bar: 10 μm.
(TIF)

S3 Fig Endogenous GAD65 in human pancreatic β-cells is primarily targeted to the Golgi compartment and to vesicles distinct from insulin secretory vesicles. Projected confocal images of human islet single cells imaged at 40nm per pixel resolution (A) or 100 nm per pixel resolution (B) and immunostained for endogenous GAD65 (green, GAD6 antibody), insulin (magenta), the Golgi marker protein giantin (red), and the nuclear stain DAPI (blue). In human islet single cells, GAD65 is expressed in Golgi membranes (enlarged frames, lower left panels) and cytosolic vesicles that are distinct from insulin containing vesicles (enlarged frames, lower middle panels). Scale bar: 10 μm.
(TIF)

S4 Fig Expression of GAD65 in rat islet cells is restricted to insulin positive β-cells and not detected in glucagon-positive α-cells. Projected confocal images of rat islet single cells immunostained for endogenous GAD65 (GAD6 antibody, green), insulin (red), and glucagon (magenta). GAD65 expression is confined to insulin positive β-cells (red) and not detected in the glucagon-positive α-cell (magenta). The arrowhead indicates an insulin positive cell that is GAD65 negative. Scale bar: 10 μm.
(TIF)

S5 Fig NAP22 is expressed in rat islets, rat brain and INS-1 cells. Immunoblotting analysis of endogenous expression of NAP22 in lysates of rat islets (lane 1), rat brain homogenate (lane 2) and INS-1 cells (lane 3). Equal amounts of protein (10 μg) were loaded in each lane. NAP22 is expressed in all three cell types/tissues.
(TIF)

S6 Fig Confocal analyses of NAP22 expression and subcellular distribution in INS-1 cells, rat islets cells, and neurons reveal minor or no colocalization with GAD67 and GAD65. (A) Projected confocal images of INS-1 cells singly transfected with mGAD67-GFP and immunostained for GFP (green) and NAP22 (red). (B) Projected confocal images of rat pancreatic islet cells immunostained for endogenous GAD65 (GAD6 antibody, green) and NAP22 (red). In both cell types, NAP22 is mainly detected in the plasma membrane and colocalization between NAP22 and either GAD67 or GAD65 GAD is either non-existent or minimal. Scale bar: 10 μm. (C) Projected confocal images of hippocampal neurons immunostained for NAP22 (NAP22 antibody, red) and endogenous GAD65 (GAD6 antibody, green). In some axonal areas, no colocalization between NAP22 and GAD65 is detected (enlarged frame *a*), while is other axonal areas, co-localization between NAP22 and GAD65 is observed in a small fraction of axonal boutons (enlarged frame *b*). Scale bar: 30 μm (C) and 10 μm (*a* and *b*).
(TIFF)
