# Supplementary figures and images for: Compartmentalization of GABA Synthesis by GAD67 Differs between Pancreatic Beta Cells and Neurons
Source: PLoS One. 2015 Feb 3;10(2):e0117130. doi: 10.1371/journal.pone.0117130 (PMC4315522; doi:10.1371/journal.pone.0117130)

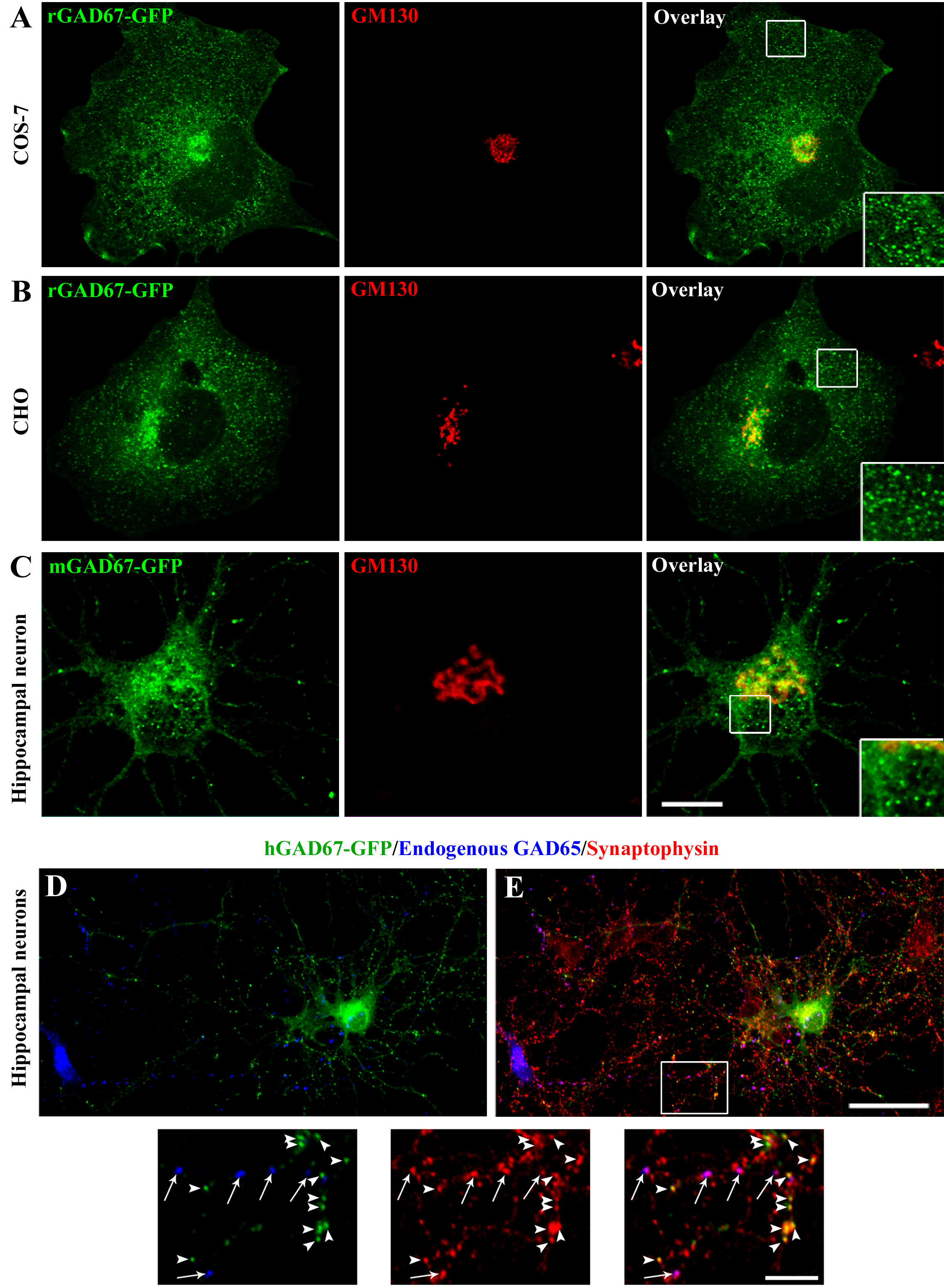

Supplement: S1 Fig — Projected confocal images of a COS-7 cell (A), CHO cell (B), and rat hippocampal neurons (C-E) transiently expressing GAD67-GFP. COS-7 and CHO cells were fixed 24 h following transfection. Rat hippocampal neurons were transfected at DIV 6 and fixed 72 h following transfection. (A-C) Cells were immunostained for GFP (green) and the Golgi marker GM130 (red). GAD67-GFP is targeted to Golgi membranes and cytosolic vesicles (A-C, enlarged frames) in COS-7 cells (A), CHO cells (B), and rat hippocampal neurons (C). (D-E) Rat hippocampal neurons were triple immunostained for GFP (green), synaptophysin (red), and endogenous GAD65 (GAD6 antibody, blue). The transfected neuron is a non-GABAergic neuron devoid of endogenous GAD65. However, GAD65 is seen in the cell body and axonal puncta of a neighboring GABAergic neuron (blue). Human GAD67-GFP colocalizes with synaptophysin in presynaptic clusters (arrowheads, enlarged frame), which are devoid of GAD65. Endogenous GAD65 colocalizes with synaptophysin in presynaptic clusters of the non-transfected GABAergic neuron in the same field of view (arrows, enlarged frame). Scale bars: 10 μm. (TIF) [file pone.0117130.s001.tif]

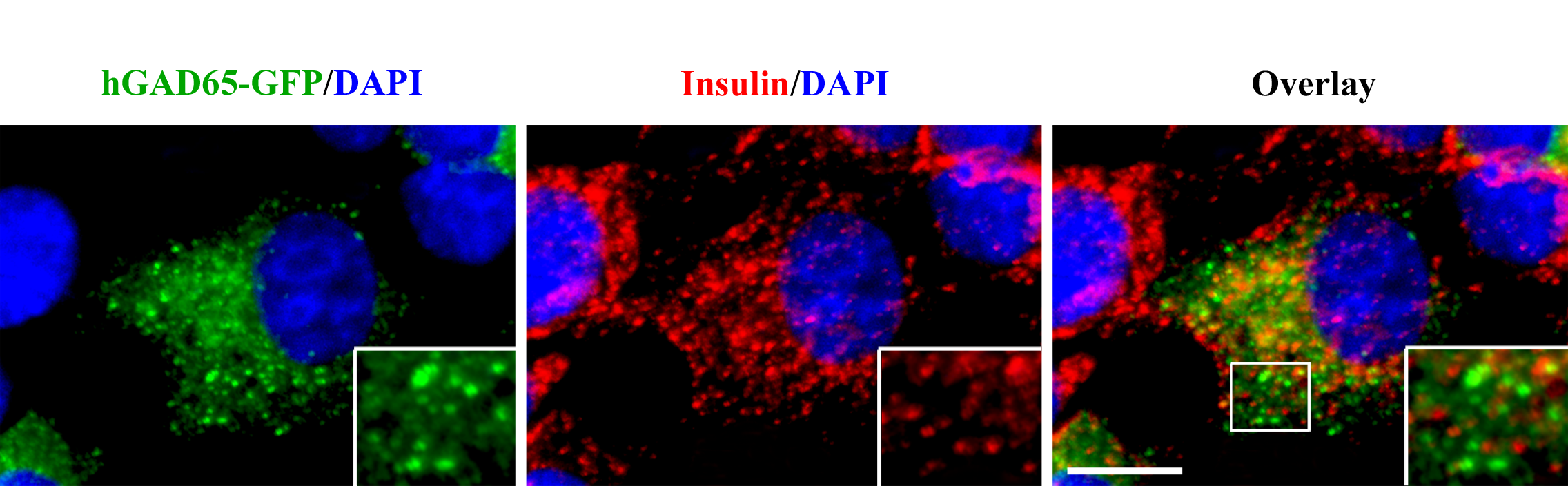

Supplement: S2 Fig — Projected confocal images of INS-1 cells singly transfected with hGAD65-GFP and immunostained for GFP (green), endogenous insulin (red) and the nuclear stain DAPI (blue). GAD65-GFP-containing vesicles do not co-localize with insulin-containing large dense core vesicles (enlarged frame). Scale bar: 10 μm. (TIF) [file pone.0117130.s002.tif]

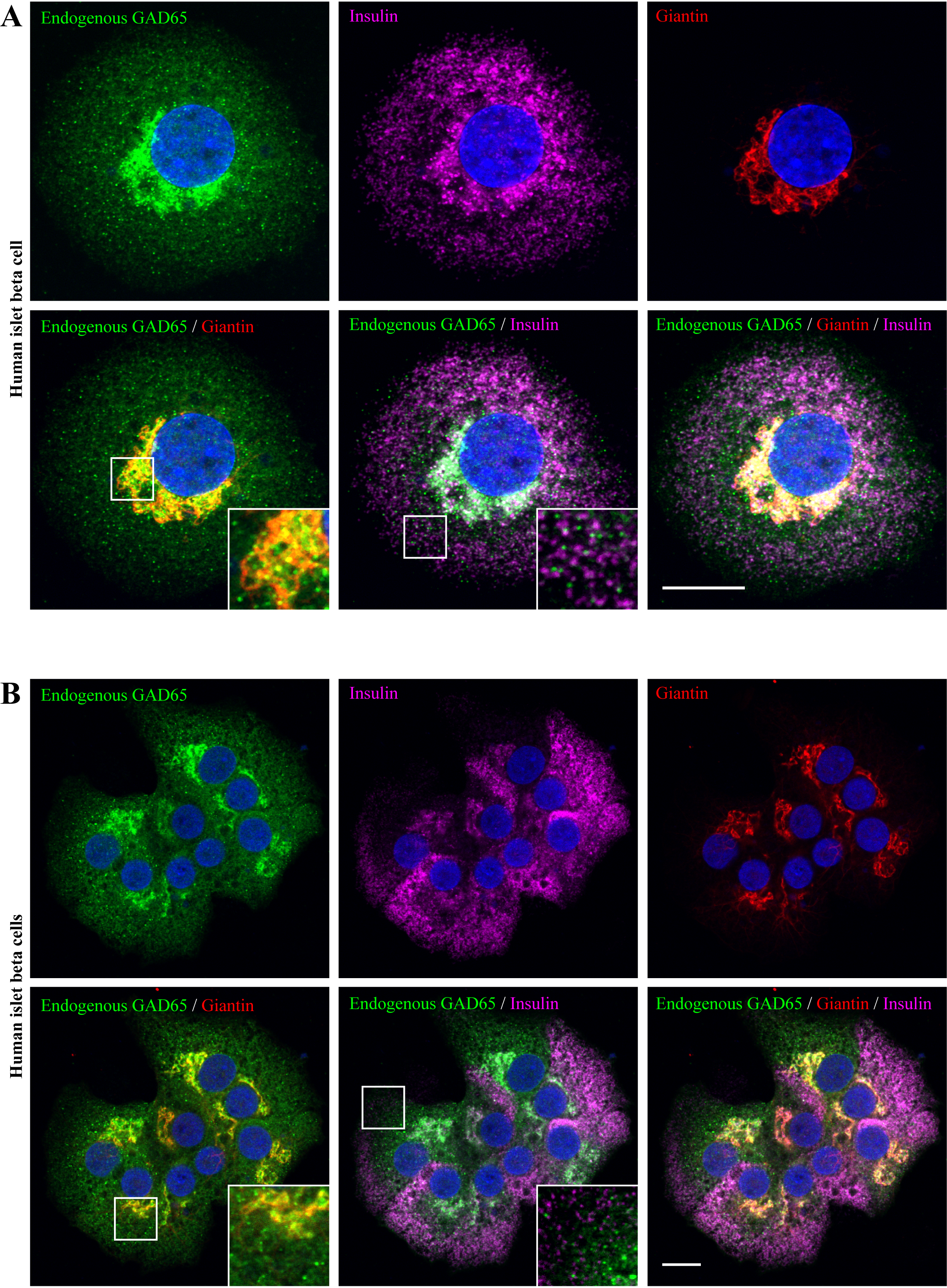

Supplement: S3 Fig — Projected confocal images of human islet single cells imaged at 40nm per pixel resolution (A) or 100 nm per pixel resolution (B) and immunostained for endogenous GAD65 (green, GAD6 antibody), insulin (magenta), the Golgi marker protein giantin (red), and the nuclear stain DAPI (blue). In human islet single cells, GAD65 is expressed in Golgi membranes (enlarged frames, lower left panels) and cytosolic vesicles that are distinct from insulin containing vesicles (enlarged frames, lower middle panels). Scale bar: 10 μm. (TIF) [file pone.0117130.s003.tif]

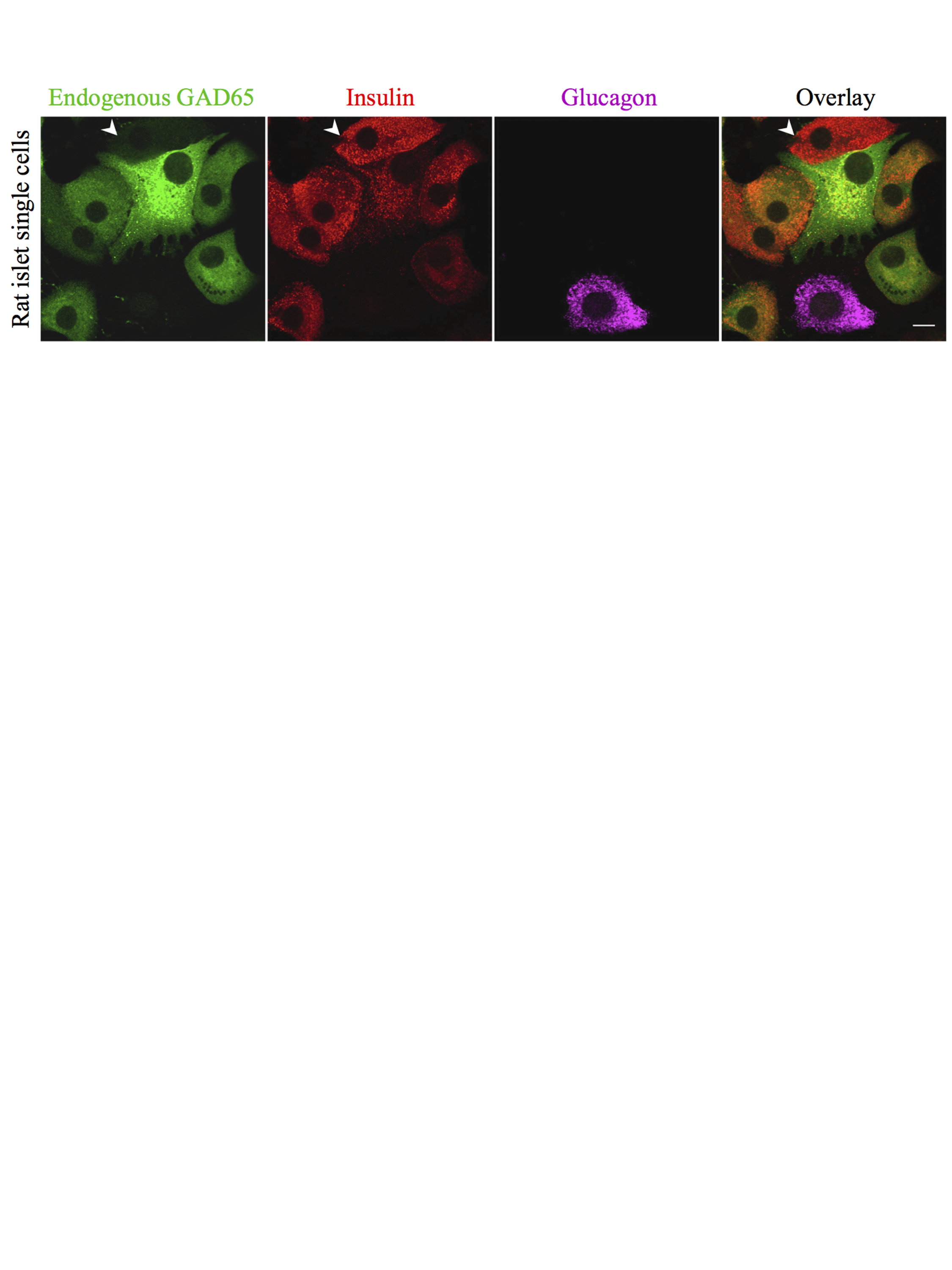

Supplement: S4 Fig — Projected confocal images of rat islet single cells immunostained for endogenous GAD65 (GAD6 antibody, green), insulin (red), and glucagon (magenta). GAD65 expression is confined to insulin positive β-cells (red) and not detected in the glucagon-positive α-cell (magenta). The arrowhead indicates an insulin positive cell that is GAD65 negative. Scale bar: 10 μm. (TIF) [file pone.0117130.s004.tif]

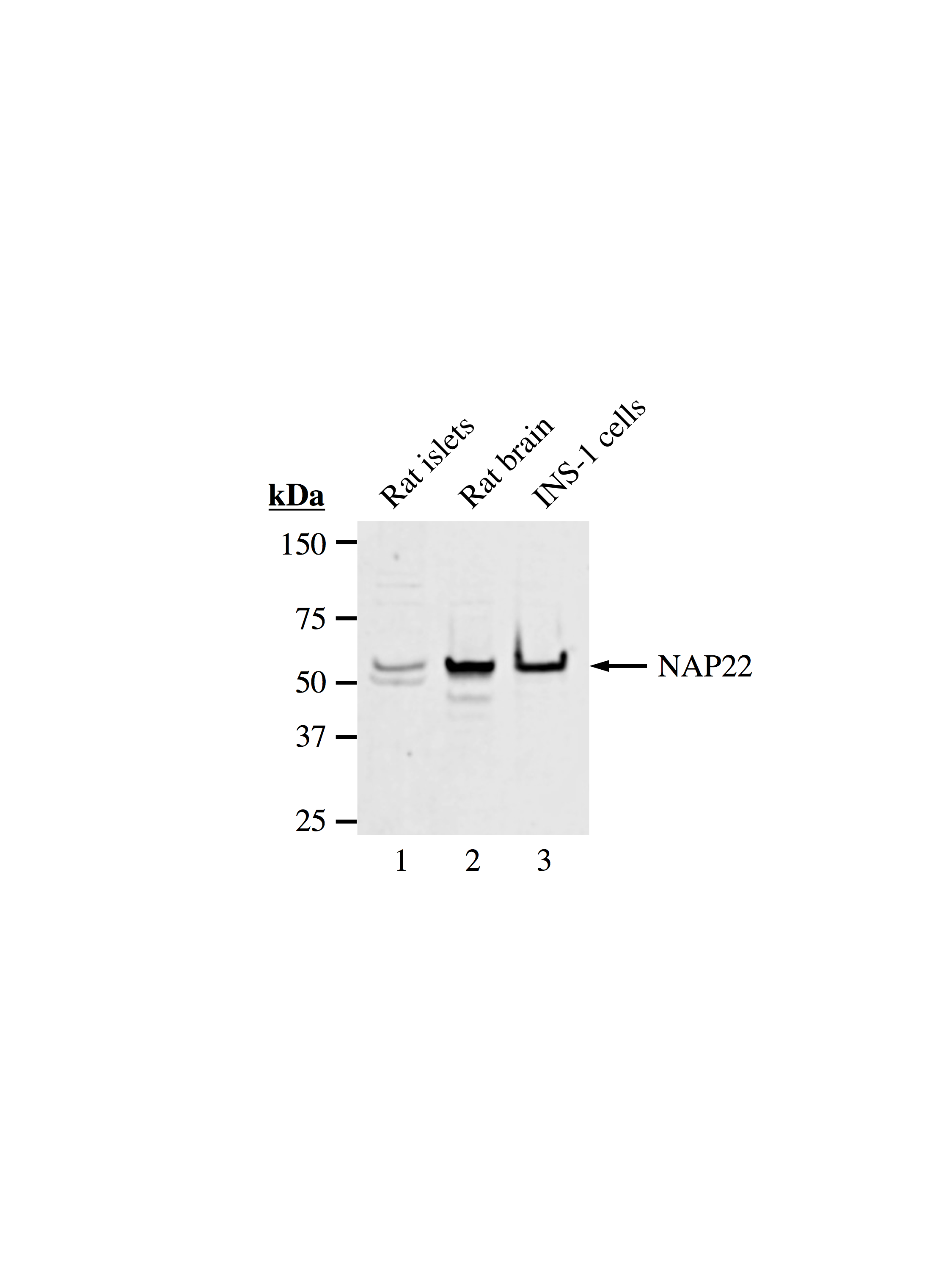

Supplement: S5 Fig — Immunoblotting analysis of endogenous expression of NAP22 in lysates of rat islets (lane 1), rat brain homogenate (lane 2) and INS-1 cells (lane 3). Equal amounts of protein (10 μg) were loaded in each lane. NAP22 is expressed in all three cell types/tissues. (TIF) [file pone.0117130.s005.tif]

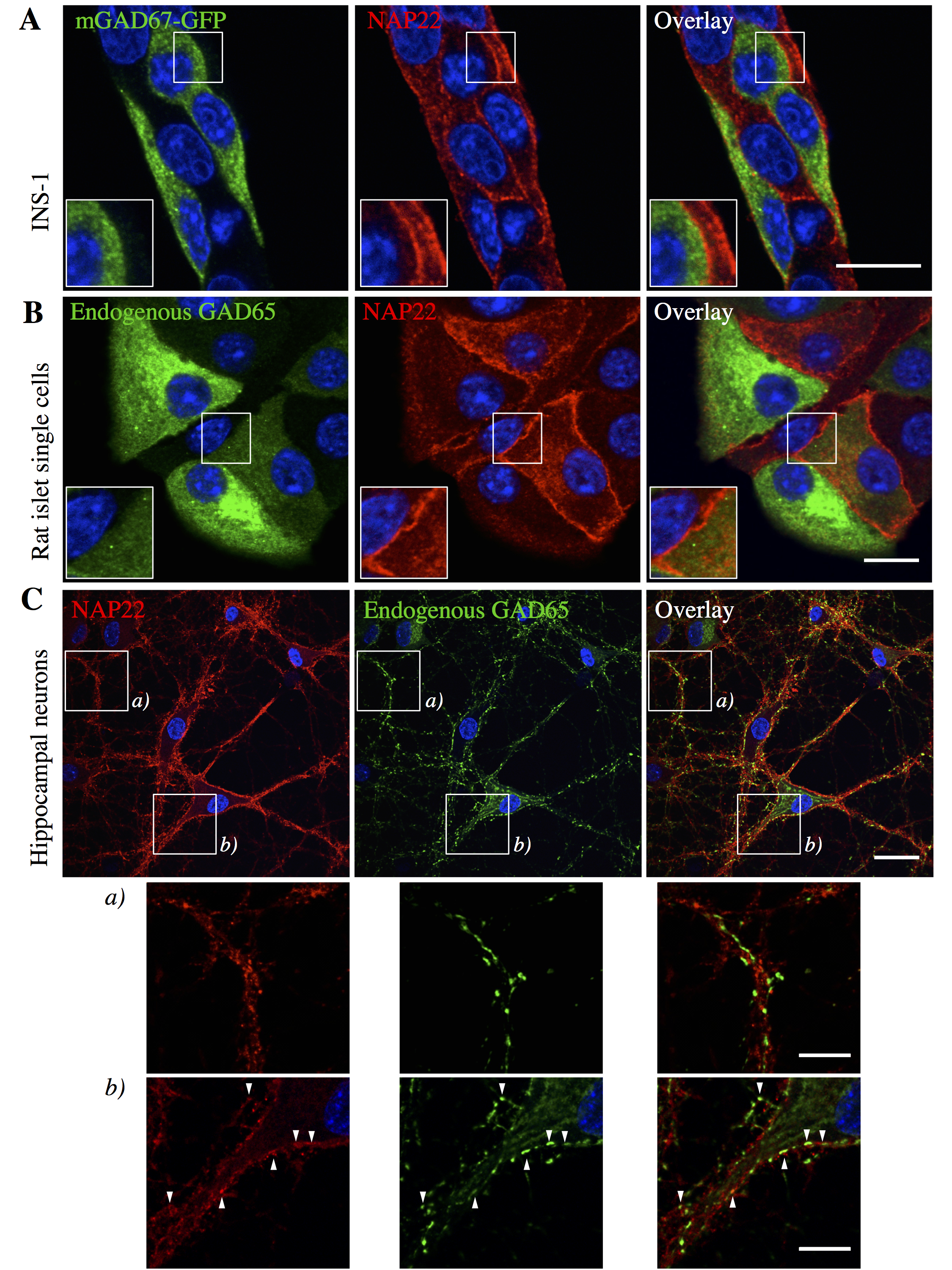

Supplement: S6 Fig — (A) Projected confocal images of INS-1 cells singly transfected with mGAD67-GFP and immunostained for GFP (green) and NAP22 (red). (B) Projected confocal images of rat pancreatic islet cells immunostained for endogenous GAD65 (GAD6 antibody, green) and NAP22 (red). In both cell types, NAP22 is mainly detected in the plasma membrane and colocalization between NAP22 and either GAD67 or GAD65 GAD is either non-existent or minimal. Scale bar: 10 μm. (C) Projected confocal images of hippocampal neurons immunostained for NAP22 (NAP22 antibody, red) and endogenous GAD65 (GAD6 antibody, green). In some axonal areas, no colocalization between NAP22 and GAD65 is detected (enlarged frame a), while is other axonal areas, co-localization between NAP22 and GAD65 is observed in a small fraction of axonal boutons (enlarged frame b). Scale bar: 30 μm (C) and 10 μm (a and b). (TIFF) [file pone.0117130.s006.tiff]
